# 196. Repeat Tracheal Aspirates in Pediatric Intensive Care Patients: Frequency, Resistance and Antimicrobial Use

**DOI:** 10.1093/ofid/ofac492.274

**Published:** 2022-12-15

**Authors:** Edward Lyon, Jennifer Goldman, Brian R Lee, Rangaraj Selvarangan, Elizabeth Monsees

**Affiliations:** Children's Mercy Hospital, Kansas City, Missouri; Children's Mercy Hospital, Kansas City, Missouri; Children's Mercy Kansas City, Kansas City, Missouri; Children's Mercy, Leawood, Kansas; Children's Mercy Kansas City, Kansas City, Missouri

## Abstract

**Background:**

Tracheal aspirates (TA) are frequently obtained in the pediatric intensive care unit (PICU); however, no data exists on the frequency or clinical management of multiple TA cultures on the same PICU patient. In this study, we describe the frequency of repeat TAs in PICU patients, the emergence of multidrug resistant organisms (MDRO), frequently cultured pathogens of TAs, and associated antibiotic prescribing patterns.

**Methods:**

We identified 70 PICU patients between 2018-2019 who met our criteria for retrospective chart review with ≥ 2 TAs obtained during their hospitalization. The following information was collected: patient demographics, number of TAs per patient, microbiology with susceptibilities, antibiotic use, and clinical data summarizing patient condition. Descriptive statistics established the frequency and time between initial and repeat TAs, reason for collection, antibiotic exposure, and frequency of MDRO development.

**Results:**

Preliminary data on 15 patients showed 90 total TA cultures, with a median of 4 [IQR 2.5, 8] cultures per patient during their PICU stay (Figure 1). The median days between cultures was 10 [IQR 4, 26]. Most patients were < 5 years of age (n=14; 94%), male (n=13; 87%), and were admitted to the medical ICU service (n=8, 53%). Fever (46%) was the most common reason for TA collection followed by vital sign changes and secretion burden (34% and 32% respectively). A total of 133 organisms were isolated, with *Pseudomonas aeruginosa* (n=32), Methicillin-susceptible *Staphylococcus aureus* (n=16), and *Klebsiella oxytoca* (n=11) comprising the top pathogens (Figure 2). Eleven of the 15 patients (73.3%) had the same organism detected on ≥ 2 separate cultures. A total of 616 antibiotic days were prescribed with 149 (24%) antibiotic days prescribed for the TA result specifically. Six (40%) patients developed a MDRO after a median of 14.5 antibiotic days [IQR 11.75, 37.5].

**Figure 1:**
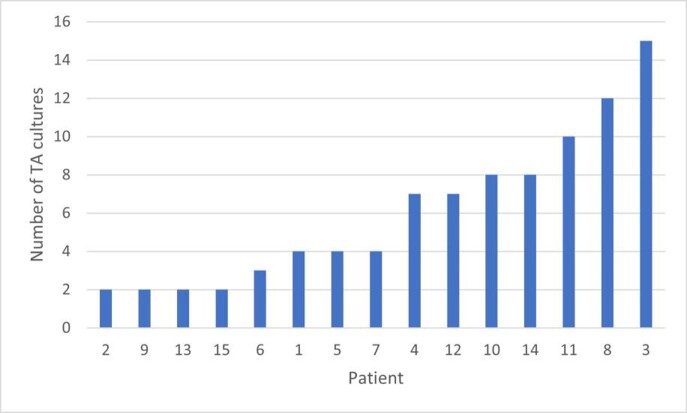
Number of tracheal aspirate cultures per patient

**Figure 2:**
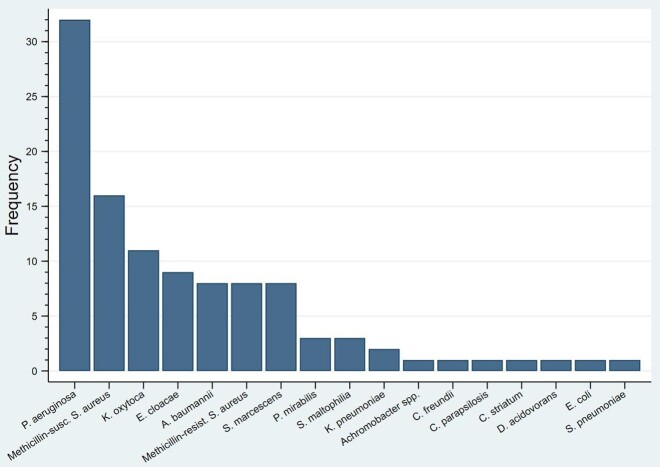
Frequency of identified pathogens

**Conclusion:**

Repeat TAs are performed in the PICU, often identifying the same pathogen repeatedly, likely representing colonization. Development of resistance is common and only one-fourth of TAs are directly treated with antibiotics. These data provide an opportunity to further explore clinical criteria to maximize the impact of TA cultures in the PICU.

**Disclosures:**

**Brian R. Lee, PhD, MPH**, CDC: Grant/Research Support|Merck: Grant/Research Support **Rangaraj Selvarangan, BVSc, PhD, D(ABMM), FIDSA, F(AAM)**, BioFire: Grant/Research Support|Luminex: Grant/Research Support.

